# Identifying Patients at Risk of Delayed Breast Imaging Due to the COVID-19 Pandemic

**DOI:** 10.7759/cureus.17235

**Published:** 2021-08-16

**Authors:** Shiyi Li, Sophia O'Brien, Christina Murphy, Calisi Nabil

**Affiliations:** 1 Interventional Radiology, Einstein Medical Center Philadelphia, Philadelphia, USA; 2 Radiology, Hospital of University of Pennsylvania, Philadelphia, USA; 3 Radiology, Perelman School of Medicine, Philadelphia, USA; 4 Radiology, Emory University School of Medicine, Atlanta, USA

**Keywords:** covid-19, breast imaging, breast cancer care, screening mammography, cancer screening, delayed care

## Abstract

Objective: Epidemiological models predict worse cancer outcomes due to COVID-19 pandemic-related delays in cancer surveillance and treatment. This study evaluated patient demographic factors associated with delayed breast imaging or procedure appointments due to COVID-19.

Methods: Patients attending a breast imaging or procedure appointment at the Pennsylvania Hospital Breast Center from December 28, 2020 to January 31, 2021 were asked to complete a voluntary and anonymous survey on the impact of COVID-19. Chi-squared and two-sample t-tests were used to analyze correlations between having a delayed appointment and various demographic variables.

Results: Five hundred seventy patients completed the survey. Participants were more likely to have delayed a breast imaging or procedure appointment if they were younger (53.9 versus 57.4 years old, p=0.014), had more total household residents (2.7 versus 2.2, p=0.019) or children (0.8 versus 0.4, p=0.016), personally had COVID-19 (p=0.04), or personally had to quarantine (p<0.01). Race, ethnicity, education, income level, and marital status were not found to statistically significantly correlate with having a delayed appointment.

Conclusion: This study found that younger age, a greater number of residents and children in the household, and having a personal history of COVID-19 infection or quarantining were factors significantly correlated with delaying a breast imaging or procedure appointment. As radiology practices prepare to mitigate the impact of COVID-19 on screening practices and cancer outcomes, these findings may help imaging centers refine patient outreach efforts and policy accommodations to protect the most vulnerable populations.

## Introduction

Declared a global pandemic in March 2020, the novel coronavirus, SARS-CoV-2, has transformed almost every aspect of healthcare in the US. With many major multidisciplinary health societies issuing guidelines to delay routine or elective appointments, the field of breast imaging and breast cancer care has experienced significant disruptions, ranging from delayed screening mammography to alterations in treatment algorithms. When studies emerged in late March 2020 demonstrating that radiology technologists were among the healthcare workers most susceptible to work-related COVID-19 infection, the American Society of Breast Surgeons and American College of Radiology quickly released a joint statement that recommended postponement of all breast screening exams and routine breast health appointments [1].

By April 2020, the COVID-19 Pandemic Breast Cancer Consortium was created to modify breast cancer triage and treatment algorithms with the goal of preserving hospital resources for COVID-19 patients without significantly compromising long-term outcomes for breast cancer patients [2]. As hospitals became better adapted to the pandemic by July 2020, breast cancer guidance documents began to ease cautions against routine appointments and encourage a return to routine screening when an imaging center could do so safely. However, many large healthcare institutions have reported profound declines in breast imaging case volume, ranging from 41% to 88%, especially at the pandemic’s height from March to July 2020 [3-6]. Additionally, many breast imaging centers continue to experience widespread appointment delays [7-10].

Few studies currently exist on the long-term impact of these delays on cancer outcomes. Experts predict that since the pandemic’s early phase saw a marked decrease in all new cancer diagnoses, likely due to patients not presenting for care, these cancers will present later, at a higher stage, and with a worse prognosis [11]. An epidemiological cancer outcome model, assuming a six-month disruption of care during the pandemic, estimated more than 10,000 additional deaths in the US in the next decade due to breast and colorectal cancer [12]. Another modeling study of cancer patients older than 30 in the United Kingdom suggested between 3,316 and 9,948 additional life-years lost, assuming that 25% to 75% of patients delayed cancer presentation until after a three-month lockdown period [13].

Elucidating the factors correlated with a greater likelihood to delay a breast care appointment due to COVID-19, therefore, became a priority to help providers identify vulnerable patient populations and implement policies to protect them from worse cancer outcomes. To our knowledge, no prior studies have characterized the patient population whose breast cancer screening was delayed due to COVID-19 or evaluated if certain patients were more likely to experience delayed care than others.

## Materials and methods

This anonymous prospective study was determined to be exempt from human subject review by the authors’ institutional review board. A 29-question paper survey, comprising a combination of multiple-choice, yes or no, and free-text questions, was distributed to all patients who attended an in-person breast imaging or procedure appointment at the authors’ breast imaging center from December 28, 2020 to January 31, 2021. Participants were asked to complete the entirety of the survey voluntarily. The authors gave participants disposable pens and left paper surveys untouched in a box for two days before data input to reduce potential COVID-19 exposure among participants and staff.

The survey consisted of four sections (see Appendix). In the first multiple-choice sections, participants were asked to provide basic demographic information such as gender, race, ethnicity, marital status, neighborhood setting, education background, the primary mode of transportation, insurance, and income level. They were additionally asked to provide numerical free-text answers to the age and number of residents and children in their households. The second section focused on participant experience during the COVID-19 pandemic. In a series of yes or no questions, participants were asked if they personally suffered from COVID-19, had household or outside family members suffer from COVID-19, or had to quarantine. They were also asked if COVID-19 impacts their ability to work, use transportation, pay rent or mortgage, receive healthcare needs or obtain insurance status. The third section focused on participants’ experience with breast imaging appointments during COVID-19. In yes or no responses, participants answered if they missed or delayed an appointment, the number of weeks by which their appointment was delayed, and their perception of screening mammography as a healthy priority before and after COVID-19. The final section inquired about personal and family history of breast and other cancers. In yes or no responses, participants indicated if they have dense breasts, a personal history of breast or other cancers, a known genetic mutation that predisposes to breast cancer, and a family history of breast cancer.

Survey responses were transcribed in Excel, and omitted answers were excluded from the analysis. Baseline demographic results were presented as a total number of participants per response and proportion within each category. The primary outcome of our analysis was to identify statistically significant correlations between specific demographic or COVID-19-related factors and a greater likelihood to delay an imaging or procedure appointment. Bivariate analysis was performed on the variable of delaying and not delaying an appointment. Chi-squared tests were used to analyze the significance of correlations between delaying an appointment and categorical variables, which included most survey responses to baseline demographic questions, the impact of COVID-19 on general life questions, and cancer history questions. Two-sample t-tests were used to analyze the significance of correlations between delaying an appointment and the numerical variables of average age, number of residents, and number of children per household. P-values less than 0.05 were considered statistically significant. Analysis was performed using Python and Excel.

## Results

Of the 1,481 patients who attended a breast imaging or procedure appointment at the author’s breast imaging center from December 28, 2020 to January 31, 2021, 570 patients responded to the survey and were included in this study (38.5% response rate). All participants were female. Of the 570 participants, 62 reported a delayed breast imaging or procedure appointment due to COVID-19 (11.2%).

Most participants were White (59.8%) or Black/African American (34.9%) and not of Hispanic, Latino, or Spanish descent (80.7%). Most participants fell within the age range of 40 to 70 years old (79.6%), lived in an urban neighborhood (75.6%), and had private insurance (58.3%, Table 1).

**Table 1:** Baseline Patient Demographics

Category	Variable	Count			Percentage
Race	White		341	59.8	
	Black or African American		199	34.9	
	Asian		15	2.6	
	Native Hawaiian or Other Pacific Islander		2	0.4	
	Omitted		13	2.3	
Ethnicity	Not Hispanic or Latino or Spanish		460	80.7	
	Hispanic or Latino or Spanish Origin		20	3.5	
	Omitted		90	15.8	
Age	20-30		4	0.7	
	30-40		10	1.8	
	40-50		167	29.3	
	50-60		145	25.4	
	60-70		142	24.9	
	70-80		92	16.1	
	80-90		7	1.2	
	90-100		1	0.2	
	Omitted		2	0.4	
Marital status	Married/civil union		265	46.5	
	Single		175	30.7	
	Divorced		73	12.8	
	Widowed		42	7.4	
	Separated		12	2.1	
	Omitted		3	0.5	
# of Residents per Household	1		155	29.0	
	2		200	35.1	
	3		99	17.4	
	4		70	12.3	
	>4		32	5.6	
	Omitted		4	0.7	
Transportation	Personal vehicle		253	44.4	
	Walked		98	17.2	
	Bus or other public transportation		96	16.8	
	Friend/family member drove		61	10.7	
	Paid car service (cab, Uber, Lyft, other)		41	7.2	
	Other		7	1.2	
	Omitted		14	2.5	
Neighborhood	Urban		431	75.6	
	Suburban		117	20.5	
	Rural		13	2.3	
	Omitted		9	1.6	
Education	Some high school		70	12.3	
	Some college		100	17.5	
	Bachelor's Degree		134	23.5	
	Associate's Degree		36	6.3	
	Postgraduate Degree		182	31.9	
	Trade/Technical/Vocational Training		29	5.1	
	Omitted		19	3.3	
Insurance	Private		332	58.3	
	Medicaid/Medicare		176	30.9	
	No insurance		5	0.9	
	Other		39	6.8	
	Omitted		18	3.2	
Income	<25,000		60	10.5	
	25,000 - <50,000		83	14.6	
	50,000 - <100,000		118	20.7	
	100,000 - <150,000		116	20.4	
	>150,000		134	23.5	
	Omitted		59	10.4	
# of Children per Household	0		387	67.9	
	1		85	14.9	
	2		59	10.4	
	3		11	1.9	
	>3		6	1.1	
	Omitted		22	3.9	

At the time of the survey, 5.1% of participants had a personal history of COVID-19 infection, 5.8% of participants had a household member who contracted COVID-19, and 24.4% of participants had a non-household family member who contracted COVID-19. 15.6% of participants had to undergo quarantine (Table 2).

**Table 2:** Impact of COVID-19 on General Domains of Life

Survey Question	Response	Count	Percentage
Did you personally have COVID?	Y	29	5.1
	N	524	91.9
	Omitted	17	3.0
Have you been tested for COVID?	Y	251	44.0
	N	304	53.3
	Omitted	15	2.6
Did anyone in your household have COVID?	Y	33	5.8
	N	522	91.6
	Omitted	15	2.6
Did anyone in your family (outside your household) have COVID?	Y	139	24.4
	N	416	73.0
	Omitted	15	2.6
Did you have to quarantine?	Y	89	15.6
	N	464	81.4
	Omitted	17	3.0
COVID affected my ability to work	Y	93	16.3
	N	462	81.1
	Omitted	15	2.6
COVID affected my ability to get healthcare	Y	23	4.0
	N	532	93.3
	Omitted	15	2.6
COVID affected my insurance status	Y	10	1.8
	N	545	95.6
	Omitted	15	2.6
COVID affected my ability to use transportation	Y	85	14.9
	N	470	82.5
	Omitted	15	2.6
COVID affected my ability to pay rent/mortgage	Y	28	4.9
	N	527	92.5
	Omitted	15	2.6

Most participants were visiting the breast imaging center for a screening mammogram appointment (85.8%). 10.4% of participants had personally had breast cancer and 18.9% had a family history of breast cancer (Table 3).

**Table 3:** Personal History of Breast Cancer Screening and Risk Factors

Survey Question	Response	Count	Percentage
Why are you here today?	Screening exam	489	85.8
	Non-screening exam	51	8.9
	Procedure	11	1.9
	Omitted	19	3.3
Have you ever been told that you have dense breasts?	Y	304	53.3
	N	246	43.1
	Omitted	20	3.5
Have you personally had breast cancer?	Y	59	10.4
	N	496	87.0
	Omitted	15	2.6
Have you personally had any cancer?	Y	68	11.9
	N	478	83.9
	Omitted	25	4.4
Do you have a genetic mutation placing you at high risk for breast cancer?	Y	4	0.7
	N	547	96.0
	Omitted	19	3.3
Do you have a family history of breast cancer?	Y	108	18.9
	N	433	76.0
	Omitted	29	5.1

The average age of participants who delayed a breast imaging or procedure appointment due to COVID-19 was lower than those who did not delay an appointment (53.9 versus 57.4 years old, p=0.014; Table 4). The average number of total residents and children living in a participant’s household was higher for participants who delayed an appointment than for those who did not (2.7 versus 2.2, p=0.019 and 0.8 versus 0.4, p=0.016, respectively). A greater percentage of participants with private insurance delayed an appointment compared to those insured by Medicaid/Medicare, approaching but not reaching statistical significance (p=0.077). Race, ethnicity, education, income level, marital status, neighborhood locality, and primary mode of transportation were not found to have a statistically significant correlation with a participant’s likelihood to delay an appointment.

**Table 4:** Correlation of Breast Imaging Appointment Delay Due to COVID-19 With Baseline Patient Demographics ^a^n=544,^b^n=543, ^c^n=472, ​​​​​​​ ^d^n=537, ^e^n=499, ^f^n=538, ^g^n=553, ^h^n=548, ^i^n=542, ^j^n=552, ^k^n=536

Did you miss or delay a breast imaging appointment or procedure due to COVID-19?				
Category	Variable	Delay (%)	No Delay (%)	P-value
Average Age^a^		53.9 ± 10.0	57.4 ± 11.8	0.014
Race^b^	White	41 (66.1)	290 (60.3)	0.79
	Black or African American	19 (30.6)	177 (36.8)	
	Asian	2 (3.2)	13 (2.7)	
	Native Hawaiian/other Pacific Islander	0 (0)	1 (0.2)	
Ethnicity^c^	Hispanic/Latino or Spanish origin	1 (1.9)	18 (4.3)	0.64
	Not Hispanic/Latino or Spanish	52 (98.1)	401 (95.7)	
Education^d^	Some high school	5 (8.5)	63 (13.2)	0.93
	Some college	10 (16.9)	88 (18.4)	
	Bachelor's degree	16 (27.1)	114 (23.8)	
	Associate's degree	4 (6.8)	31 (6.5)	
	Postgraduate degree	21 (35.6)	160 (33.5)	
	Trade/technical/vocational training	3 (5.1)	22 (4.6)	
Income^e^	<25,000	4 (6.9)	55 (12.5)	0.56
	25,000-<50,000	7 (12.1)	73 (16.6)	
	50,000-<100,000	15 (25.9)	100 (22.7)	
	100,000-<150,000	16 (27.6)	96 (21.8)	
	>150,000	16 (27.6)	117 (26.5)	
Insurance^f^	Private	43 (71.7)	282 (59.0)	0.077
	Medicaid/Medicare	11 (18.3)	160 (33.5)	
	No insurance	0 (0)	5 (1)	
	Other	6 (10)	31 (6.5)	
Marital Status^g^	Married/civil union	35 (56.5)	224 (45.6)	0.39
	Single	18 (29)	153 (31.2)	
	Divorced	6 (9.7)	64 (13)	
	Widowed	3 (4.8)	38 (7.7)	
	Separated	0 (0)	12 (2.4)	
Neighborhood^h^	Urban	47 (75.8)	375 (77.2)	0.93
	Suburban	14 (22.6)	101 (20.8)	
	Rural	1 (1.6)	10 (2.1)	
Transportation^i^	Personal vehicle	34 (55.7)	212 (44.1)	0.65
	Paid car service (cab, Uber, Lyft, other)	3 (4.9)	35 (7.3)	
	Bus or other public transportation	8 (13.1)	87 (18.1)	
	Friend/family member drove	6 (9.8)	55 (11.4)	
	Walked	9 (14.8)	86 (17.9)	
	Other	1 (1.6)	6 (1.2)	
Average # of Residents per Household^j^		2.7 ± 1.5	2.2 ± 1.2	0.019
Average # of Children per Household^k^		0.8 ± 1.0	0.4 ± 0.8	0.016

Participants who had a personal history of COVID-19 infection and those who had to quarantine were more likely to delay a breast imaging or procedure appointment (p=0.04 and 0.0005, respectively, Table 5). A greater percentage of participants whose ability to work was affected by COVID-19 delayed an appointment compared to those whose ability to work was not impacted, approaching but not reaching statistical significance (p=0.06). All other survey questions on the impact of COVID-19 on general domains of life were found to have no statistically significant correlation with a participant’s likelihood to delay an appointment.

**Table 5:** Correlation of Breast Imaging Appointment Delay Due to COVID-19 With Impact of COVID-19 on General Domains of Life ^a^n=543, ^b^n=544,^ c^n=542

Did you miss or delay a breast imaging appointment or procedure due to COVID-19?				
Survey Question	Response	Delay (%)	No Delay (%)	P-value
Did you personally have COVID-19?^a^	Y	3 (4.9)	65 (13.5)	0.04
	N	58 (95.1)	417 (86.5)	
Have you been tested for COVID-19?^b^	Y	26 (42.6)	223 (46.2)	0.7
	N	35 (57.4)	260 (53.8)	
Did anyone in your household have COVID-19?^b^	Y	5 (8.2)	28 (5.8)	0.65
	N	56 (91.8)	455 (94.2)	
Did anyone in your family (outside your household) have COVID-19?^b^	Y	16 (26.2)	119 (24.6)	0.91
	N	45 (73.8)	364 (75.4)	
Did you have to quarantine?^c^	Y	20 (32.8)	69 (14.3)	0.0005
	N	41 (67.2)	412 (85.7)	
COVID-19 affected my ability to work^b^	Y	16 (26.2)	76 (15.7)	0.06
	N	45 (73.8)	407 (84.3)	
COVID-19 affected my ability to get healthcare^b^	Y	5 (8.2)	18 (3.7)	0.19
	N	56 (91.8)	465 (96.3)	
COVID-19 affected my insurance status^b^	Y	1 (1.6)	9 (1.9)	0.7
	N	60 (98.4)	474 (98.1)	
COVID-19 affected my ability to use transportation^b^	Y	11 (18)	73 (15.1)	0.68
	N	50 (82)	410 (84.9)	
COVID-19 affected my ability to pay rent/mortgage^b^	Y	6 (9.8)	22 (4.6)	0.15
	N	55 (90.2)	461 (95.4)	

No survey responses regarding a participant’s personal history of breast cancer or cancer risk factors were found to have a significant correlation with the likelihood to delay an appointment due to COVID-19. A smaller percentage of participants who personally had cancer delayed an appointment due to COVID-19 compared to those who did not have a personal history of cancer, approaching but not reaching significance (p=0.089, Table 6).

**Table 6:** Correlation of Breast Imaging Appointment Delay Due to COVID-19 With Personal History of Breast Cancer Screening and Risk Factors ^a^n=548,^ b^n=553, ^c^n=543, ^d^n=550, ^e^n=539

Survey Question	Response	Delay (%)	No Delay (%)	p-value
Have you ever been told that you have dense breasts?^a^	Y	40 (64.5)	264 (54.3)	0.17
	N	22 (35.5)	222 (45.7)	
Have you personally had breast cancer?^b^	Y	4 (6.5)	55 (11.2)	0.36
	N	58 (93.5)	436 (88.8)	
Have you personally had any cancer?^c^	Y	3 (4.9)	65 (13.5)	0.089
	N	58 (95.1)	417 (86.5)	
Do you have a genetic mutation placing you at high risk for breast cancer?^d^	Y	0 (0)	4 (0.8)	0.93
	N	61 (100)	485 (99.2)	
Do you have a family history of breast cancer?^e^	Y	13 (21)	95 (19.9)	0.98
	N	49 (79)	382 (80.1)	

COVID-19 did not have a significant impact on participant perception of screening mammography as a healthcare priority. 87.2% of participants perceived screening mammography as a healthcare priority and 6.4% of participants did not perceive it as a priority, both prior to and after COVID-19 (Table 7).

**Table 7:** Impact of COVID-19 on Patient’s Response to if Screening Mammography Is a Healthcare Priority

		Count	Percentage
Response Changed	Y to N	15	2.7
	N to Y	20	3.6
Response Not Changed	Y and Y	478	87.2
	N and N	35	6.4

## Discussion

As the dominant health concern worldwide, the COVID-19 pandemic has caused widespread delay and avoidance of medical care within the US. In a recent nationwide survey conducted by the Center for Disease Control (CDC), 40.9% of US adults avoided medical care during the pandemic, and 31.5% avoided routine care [14,15]. Breast imaging centers, whose case volume is heavily dependent on routine appointments, have reported profoundly decreased volumes during the pandemic [3-6]. With several epidemiological health models predicting worse cancer outcomes due to pandemic-related delays in cancer surveillance and treatment, it is imperative to identify patients most vulnerable to the impact of COVID-19 and implement policy to accommodate their cancer surveillance and care needs.

Our study found that 26.3% of survey participants at the authors’ breast imaging center experienced delayed breast imaging care due to COVID-19 and that specific factors including younger age, more household residents, personally having COVID-19, and personally having to quarantine were correlated with delayed breast imaging and procedure appointments.

The external validity of this survey-based study is bolstered by parallels between the demographic distribution of the study sample and that of Philadelphia County, where the majority of the study's participants reside. Most recent 2019 data from the U.S. Census Bureau reports Philadelphia County’s racial breakdown to be 44.8% White, 43.6% Black or African American, and 7.8% Asian [16,17]. In comparison, the racial breakdown of the study’s sample is 59.8% White, 34.9% Black or African American, and 2.6% Asian. The average number of residents per household and percent of residents in urban neighborhoods is comparable between that reported by the Census Bureau for Philadelphia County (2.55 and 79%, respectively) and that found in the study (2.31 and 75.6%, respectively) [16]. 5.1% of participants in this study personally suffered from COVID, similar to an estimate of 7.2% average COVID-19 positivity rate in Philadelphia County throughout January 2021 [18,19]. Finally, NIH estimates for percent of women with BI-RADS category C or D breasts and percent of women to be diagnosed with breast cancer within their lifetime in the U.S. (50% and 12.9%, respectively) are similar to that found in the study (53.3% and 10.4%, respectively) [18].

Participants who were statistically more likely to have a delayed breast imaging or procedure appointment were, on average, younger and had more residents and children living in their household. Given the increased risk of breast cancer with age, older participants may be more incentivized to attend routine screening appointments. The greater likelihood to have a delayed appointment among participants with more residents per household aligns with the CDC’s finding that the prevalence of medical care avoidance was significantly higher among unpaid caregivers for adults and children [14]. Conversely, no correlation was observed between a higher likelihood to have a delayed appointment with race, ethnicity, insurance education, income, marital status, and neighborhood. These findings align with that of a recent survey-based study on breast cancer treatment delays due to COVID-19, which revealed that race, ethnicity, and insurance had no impact on a participant’s likelihood to delay cancer treatment [9].

Although COVID-19 impacted daily life in many ways, only participants who had a personal history of COVID-19 infection or those who had to quarantine were more likely to have a delayed breast imaging or procedure appointment.

This study has several limitations. First, the survey was distributed only to patients who were able to attend an in-person appointment at the authors’ imaging center. The lack of representation of patients who could not attend an in-person appointment may skew our demographic and not fully reflect more vulnerable patient populations. The association of the authors' imaging center to a tertiary-care hospital may also limit the study's generalizability to elsewhere in the country. Future studies may design surveys to be delivered electronically or over the phone to capture patients who had a missed breast imaging appointment due to COVID-19 and who have yet to reschedule and attend that appointment. Second, the survey data was collected half a year after the pandemic’s height and thus is subject to recall and response bias. Finally, certain survey questions, such as “did you have to quarantine” and “affected ability to work” may have been subject to individual interpretation.

## Conclusions

In summary, 11.2% of survey participants at our breast imaging center experienced delayed breast imaging care due to COVID-19. This study revealed that younger age, a greater number of residents and children in the household, and having a personal history of COVID-19 infection or quarantining were factors significantly correlated with delaying a breast imaging or procedure appointment. As radiology practices prepare to mitigate the impact of COVID-19 on screening practices and cancer outcomes, these findings may help imaging centers refine patient outreach efforts and policy accommodations to protect the most vulnerable populations.

## Figures and Tables

**Figure 1:**
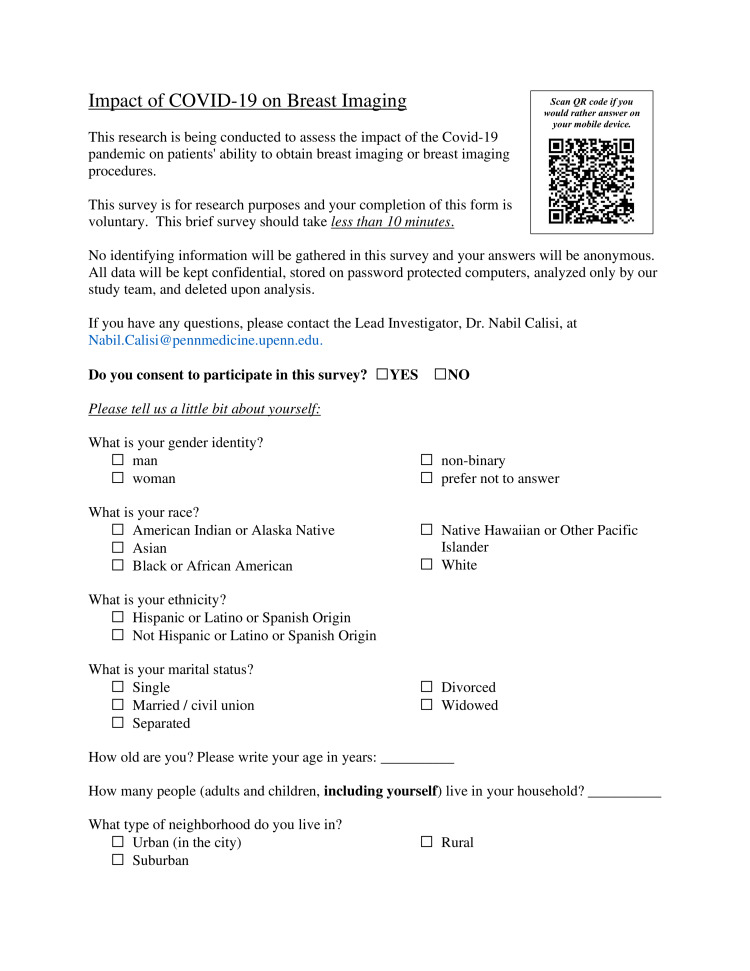
Survey Page 1

**Figure 2:**
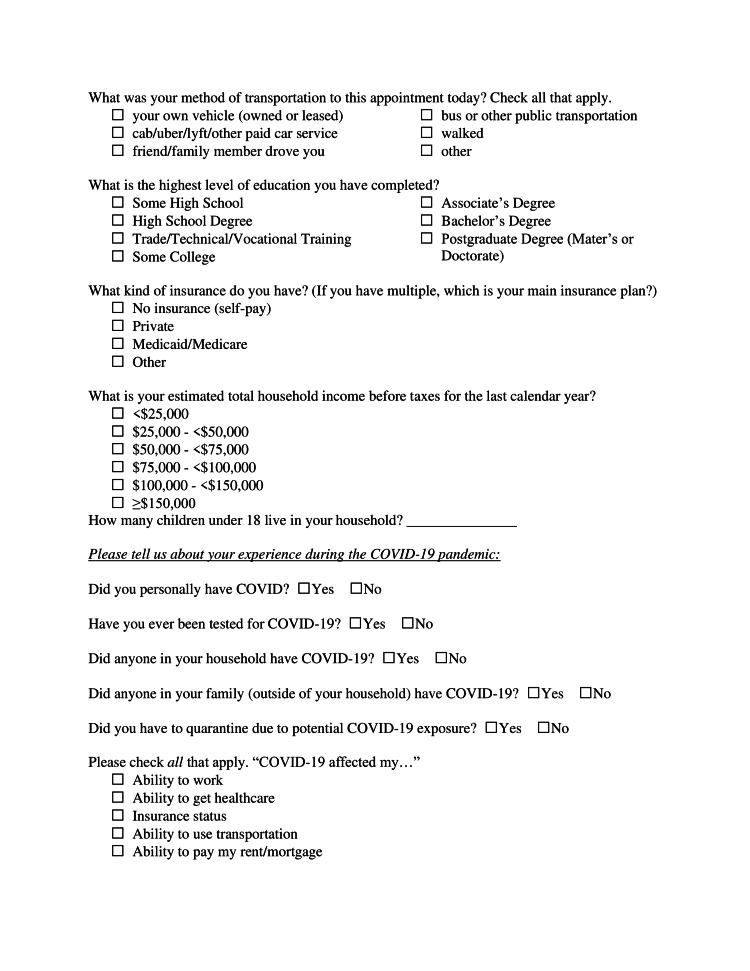
Survey Page 2

**Figure 3:**
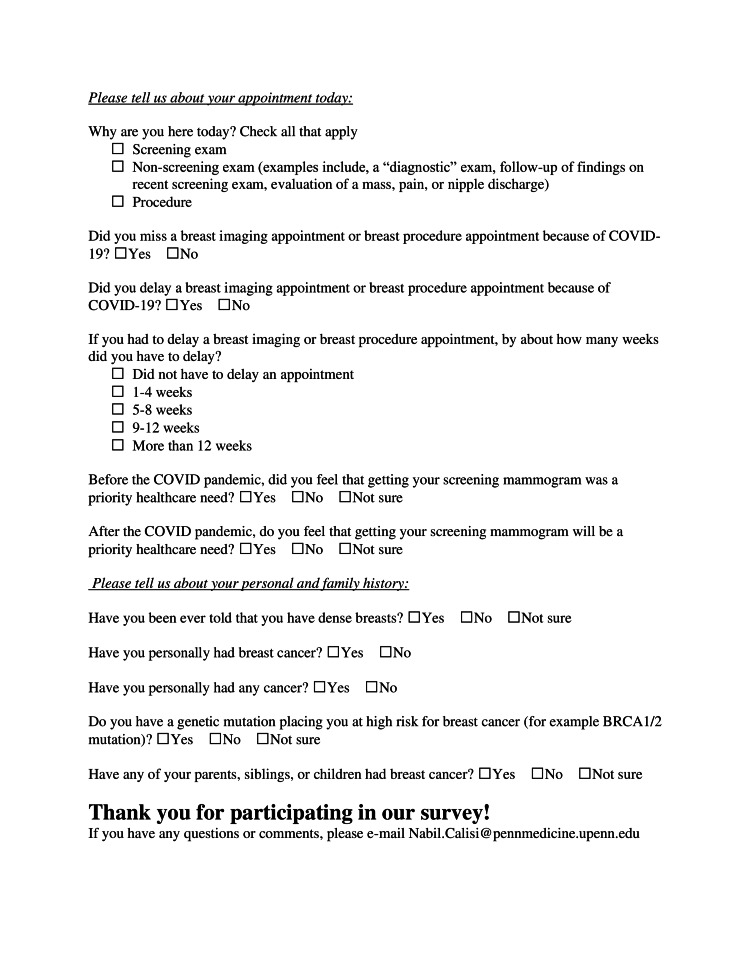
Survey Page 3

## References

[1] (2020). ASBrS and ACR Joint Statement on Breast Screening Exams During the COVID-19 Pandemic. https://www.sbi-online.org/Portals/0/Position%20Statements/2020/society-of-breast-imaging-statement-on-breast-imaging-during-COVID19-pandemic.pdf.

[2] Dietz JR, Moran MS, Isakoff SJ (2020). Recommendations for prioritization, treatment, and triage of breast cancer patients during the COVID-19 pandemic. The COVID-19 pandemic breast cancer consortium. Breast Cancer Res Treat.

[3] Naidich JJ, Boltyenkov A, Wang JJ, Chusid J, Hughes D, Sanelli PC (2020). Impact of the coronavirus disease 2019 (COVID-19) pandemic on imaging case volumes. J Am Coll Radiol.

[4] Peng SM, Yang KC, Chan WP (2020). Impact of the COVID-19 pandemic on a population-based breast cancer screening program. Cancer.

[5] Collado-Mesa F, Kaplan SS, Yepes MM, Thurber MJ, Behjatnia B, Kallos NPL (2020). Impact of COVID-19 on breast imaging case volumes in South Florida: a multicenter study. Breast J.

[6] Sendur Sendur, H. N (2020). Impacts of Covıd-19 pandemıc on breast imagıng examınatıons. EJMI.

[7] Kuderer NM, Choueiri TK, Shah DP (2020). Clinical impact of COVID-19 on patients with cancer (CCC19): a cohort study. Lancet.

[8] Freer PE (2021). The impact of the COVID-19 pandemic on breast imaging. Radiol Clin North Am.

[9] Papautsky EL, Hamlish T (2020). Patient-reported treatment delays in breast cancer care during the COVID-19 pandemic. Breast Cancer Res Treat.

[10] Yin K, Singh P, Drohan B, Hughes KS (2020). Breast imaging, breast surgery, and cancer genetics in the age of COVID-19. Cancer.

[11] Kaufman HW, Chen Z, Niles J (2020). Changes in the number of US patients with newly identified cancer before and during the coronavirus disease 2019 (COVID-19) pandemic. JAMA Netw Open.

[12] Sharpless NE (2020). COVID-19 and cancer. Science.

[13] Sud A, Torr B, Jones ME (2020). Effect of delays in the 2-week-wait cancer referral pathway during the COVID-19 pandemic on cancer survival in the UK: a modelling study. Lancet Oncol.

[14] Czeisler MÉ, Marynak K, Clarke KE (2020). Delay or avoidance of medical care because of COVID-19-related concerns - United States, June 2020. MMWR Morb Mortal Wkly Rep.

[15] Vanni G, Materazzo M, Pellicciaro M (2020). Breast cancer and COVID- 19: the effect of fear on patients' decision-making process. In Vivo.

[16] (2019). U.S. Census Bureau QuickFacts: Philadelphia County, Pennsylvania. https://www.census.gov/quickfacts/fact/table/philadelphiacountypennsylvania/PST045219.

[17] (2020). U.S. COVID Risk & Vaccine Tracker. Covid Act Now. https://covidactnow.org/us/pennsylvania-pa/county/philadelphia_county/.

[18] (2020). Dense Breasts: Answers to Commonly Asked Questions - National Cancer Institute. https://www.cancer.gov/types/breast/breast-changes/dense-breasts.

[19] U.S. Cancer Statistics Working Group (2021). USCS Data Visualizations. Centers for Disease Control and Prevention.

